# Pulmonary Smooth Muscle Hyperplasia Difficult to Differentiate from Primary Lung Cancer: A Case Report

**DOI:** 10.70352/scrj.cr.25-0428

**Published:** 2025-08-28

**Authors:** Masaya Yamasaki, Yasuaki Kubouchi, Toho Wada, Wakako Fujiwara, Shinji Matsui, Yugo Tanaka

**Affiliations:** Department of Surgery, Division of General Thoracic Surgery, and Breast and Endocrine Surgery Faculty of Medicine, Tottori University, Yonago, Tottori, Japan

**Keywords:** smooth muscle hyperplasia, lung cancer, benign pulmonary tumor

## Abstract

**INTRODUCTION:**

Pulmonary smooth muscle hyperplasia (SMH) is a rare benign tumor that presents CT imaging findings that require differentiation from those of primary lung cancer.

**CASE PRESENTATION:**

The postoperative follow-up chest CT for gastric cancer in a 76-year-old Japanese man revealed an abnormal shadow. A 2.2-cm nodule with an unclear border and showing a tendency to grow was detected in the right lower lobe (S6), and suspected infiltration into the right upper lobe (S2). PET showed minimal accumulation of 18F-fluorodeoxyglucose in the nodule, with a maximum standardized uptake value of 1.0. A transbronchial lung biopsy showed no malignant findings. Due to the tumor’s progressive growth, surgical resection was performed. Intraoperatively, a tumor located in S6 with suspected partial invasion into S2 was observed, and a wedge resection from S6 to S2 was thus performed. A frozen section of the resected specimen revealed irregularly distributed atypical cells forming mildly irregular glandular structures, leading to a diagnosis of “suspected adenocarcinoma.” Robotic-assisted thoracoscopic surgery for a right S6 segmentectomy with combined wedge resection of S2 was performed. However, the final histopathological examination revealed spindle-shaped smooth muscle cells’ proliferation. The immunohistochemical analysis revealed positivity for α-SMA, desmin, and h-caldesmon, leading to a diagnosis of pulmonary SMH.

**CONCLUSIONS:**

SMH is an extremely rare benign disease that can mimic lung cancer and may be considered among the possible differential diagnoses of solitary pulmonary nodules. A careful treatment strategy, including the choice of surgical procedure, is recommended to minimize the possibility of overtreatment.

## Abbreviations

α-SMA
: alpha smooth muscle actin
FDG
: fluorodeoxyglucose
FEV_1_
: a forced expiratory volume in one second
FVC
: a forced vital capacity
h-caldesmon
: heavy caldesmon
RATS
: robotic-assisted thoracoscopic surgery
SMH
: smooth muscle hyperplasia
SRIF
: smoking-related interstitial fibrosis
SUVmax
: a maximum standardized uptake value
VC
: a vital capacity

## INTRODUCTION

Pulmonary SMH is a rare benign tumor that is characterized by the hyperplastic proliferation of smooth muscle bundles forming small nodules. We describe a case of pulmonary SMH that was difficult to distinguish from primary lung cancer.

## CASE PRESENTATION

**Fig. 1:**
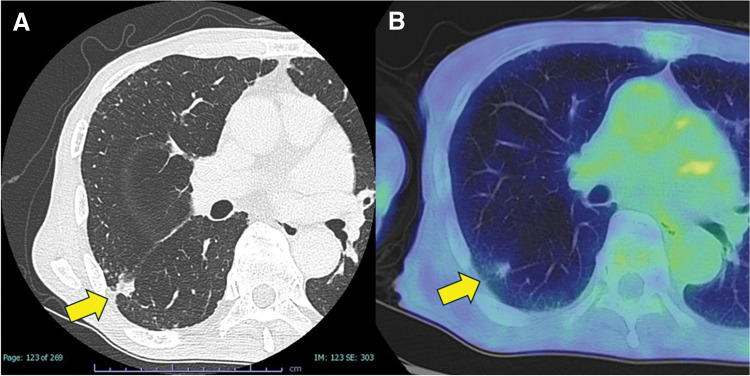
(**A**) A chest CT scan revealed a 2.2 × 1.4-cm nodule in the right lower lobe (S6) with an unclear border and a tendency to grow, plus suspected infiltration into the right upper lobe (S2). Features consistent with malignancy, such as pleural indentation and focal pleural thickening, were observed. (**B**) PET showed a minimal accumulation of FDG in the nodule, with the SUVmax of 1.0 in the early phase.

The postoperative follow-up chest CT examination for the gastric cancer of a 76-year-old Japanese man (a current tobacco smoker with a smoking history of >20 pack-years) revealed an abnormal shadow. A chest CT scan then revealed a 2.2 × 1.4-cm nodule in the right lower lobe (S6) with an unclear border and a tendency to grow, plus suspected infiltration into the right upper lobe (S2) (**Fig. 1A**). In addition, features consistent with malignancy, such as pleural indentation and focal pleural thickening, were observed. PET showed minimal accumulation of 18F- FDG in the nodule, with SUVmax of 1.0 in the early phase (**Fig. 1B**). Although a transbronchial lung biopsy revealed no evidence of malignancy, the nodule continued to grow over time, leading to the suspicion of malignancy; a decision was made to perform surgery for the nodule. Preoperative pulmonary function testing revealed VC of 2.37 L (66.4% of predicted), FVC of 2.38 L, and FEV_1_ of 2.38 L (91.9% of predicted), with an FEV1/FVC ratio of 100%, indicating mildly reduced lung volume but preserved airflow. Because it was difficult to distinguish between a benign and malignant tumor based on imaging findings and clinical course, we decided to determine the surgical procedure intraoperatively with the aid of frozen section analysis. If the lesion was found to be malignant, we planned to perform a segmentectomy as a passive limited resection, considering the patient’s pulmonary function and age. If the lesion was benign, a wedge resection would be sufficient.

**Fig. 2:**
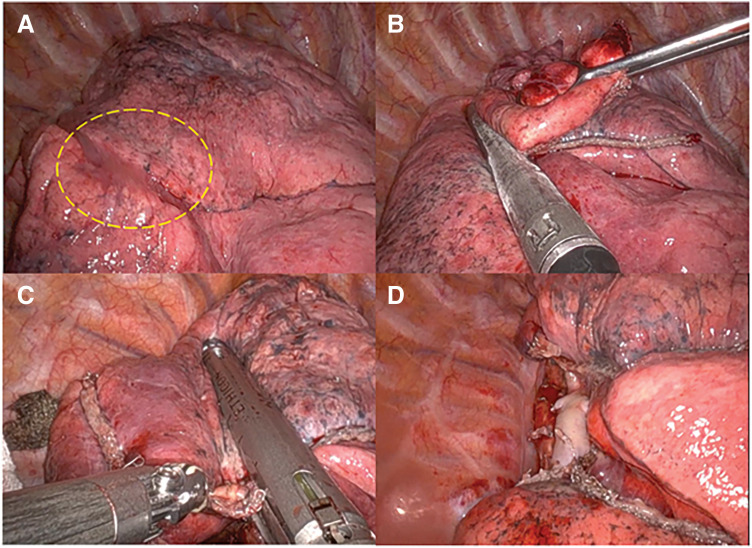
(**A**) A tumor located in the right lower lobe (S6) with suspected partial invasion into the right upper lobe (S2) was observed. (**B**) A wedge resection from S6 to S2 was thus performed. (**C**, **D**) RATS for a right S6 segmentectomy with a combined wedge resection of S2 was performed.

**Fig. 3:**
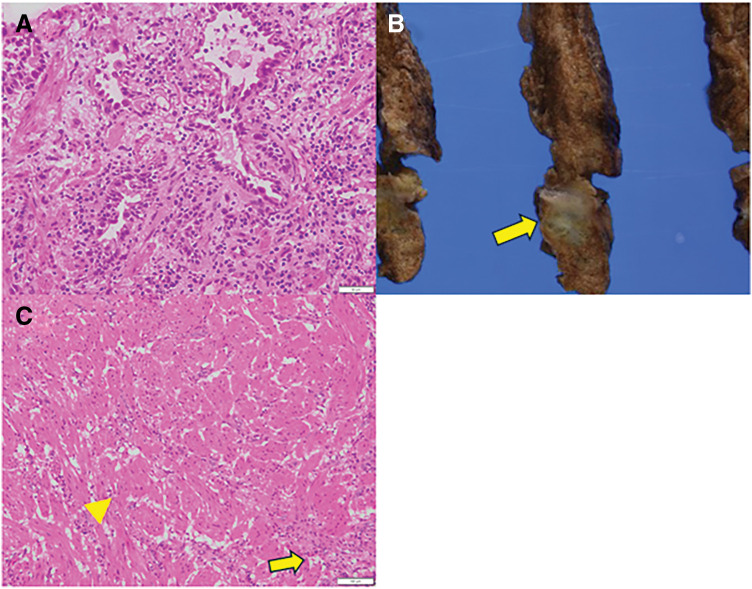
(**A**) A frozen section of the resected specimen revealed irregularly distributed atypical cells forming mildly irregular glandular structures, leading to a diagnosis of suspected adenocarcinoma; however, postoperative examination revealed that these structures represented hyperplasia of type II pneumocytes (hematoxylin and eosin stain, ×200). (**B**) Macroscopic findings of the resected specimen showed a grayish-white lesion with an unclear border. (**C**) Histological image showing both smooth muscle proliferation and hyperplasia of type II pneumocytes (hematoxylin and eosin stain, ×100). Smooth muscle proliferation is indicated by the yellow arrowhead, and hyperplasia of type II pneumocytes is indicated by the yellow arrow. Normal smooth muscle proliferation was scattered, and no malignant features were observed. These glandular structures were interpreted as hyperplasia of type II pneumocytes due to inflammation.

Intraoperatively, a tumor located in S6 with suspected partial invasion into S2 was observed (**Fig. 2A**). A wedge resection from S6 to S2 was thus performed (**Fig. 2B**). A frozen section of the resected specimen revealed irregularly distributed atypical cells forming mildly irregular glandular structures (**Fig. 3A**), leading to a diagnosis of “suspected adenocarcinoma.” RATS for a right S6 segmentectomy with a combined wedge resection of S2 was performed (**Fig. 2C** and **2D**). The patient was discharged on POD 9 without complications.

Macroscopic findings of the resected specimen revealed a grayish-white lesion with an unclear border (**Fig. 3B**). The histopathological examination showed spindle-shaped cells with eosinophilic cytoplasm forming thick fascicles. Normal smooth muscle proliferation was scattered, and no malignant features were observed (**Fig. 3C**). The immunohistochemical analysis revealed positivity for α-SMA, desmin, and h-caldesmon, leading to a diagnosis of pulmonary SMH. Although the tumor appeared to extend from S6 to S2, smooth muscle proliferation was observed separately under the visceral pleura in each segment. Since the lesion was benign and completely resected, the patient was followed without additional treatment. There is no established consensus regarding the optimal follow-up interval for such cases. Given that the lesion was a benign nodule, we plan to monitor the patient every 6 to 12 months for several years, provided that no evidence of lesion progression is observed.

## DISCUSSION

Pulmonary SMH is a rare benign lesion characterized by the hyperplastic proliferation of smooth muscle bundles forming small nodules.^1,2)^ Histologically, SMH is composed of spindle-shaped smooth muscle cells arranged in fascicles, and it typically expresses markers such as α-SMA, desmin, and h-caldesmon.^1–4)^ Other benign pulmonary lesions with spindle cell morphology include hamartoma, leiomyoma, and inflammatory myofibroblastic tumor. In the present case, the lesion is composed predominantly of smooth muscle without cartilage or adipose tissue, which excludes hamartoma.^2,4)^ Pulmonary leiomyoma is typically diagnosed as a metastatic lesion from uterine fibroids and was excluded in this case due to the patient being male.^2)^ Inflammatory myofibroblastic tumor was also excluded, as inflammatory cell infiltration was observed, but no proliferation of myofibroblasts was identified.^2,5)^ Although SMH has been reported in extrapulmonary sites such as the testicular adnexa and in association with Crohn’s disease,^6,7)^ its occurrence in the lung is extremely rare. Unlike the diffuse pulmonary smooth muscle proliferation that is observed in conditions such as asthma and pulmonary hypertension,^8)^ pulmonary SMH forming a solitary pulmonary nodule is extremely rare, and its etiology remains unclear. Possible contributing factors include chronic lung diseases such as SRIF, or scarring from prior interstitial pneumonia.^1)^ In the present case, although no radiological or pathological evidence of fibrosis was found, the patient’s long-term tobacco-smoking history may have induced chronic inflammatory changes contributing to pleural-based smooth muscle proliferation.

To date, only four instances of nodular pulmonary SMH have been documented, including our patient’s case (**Table 1**).^3,4)^ All four cases occurred in elderly individuals and involved peripherally located nodules with ill-defined margins, and the nodules were initially suspected to be malignant. These findings highlight the diagnostic challenges posed by this entity, especially in patients with risk factors such as advanced age, smoking history, or prior malignancy.

**Table 1:** Summary of the reported and present cases of pulmonary smooth muscle hyperplasia

First author	Age	Sex	Smoking history	Interstitial pneumonia	CT scan findings	Tumor size, mm	Tumor location	Surgical procedure	α-SMA	Desmin	h-caldesmon
Shikata^3)^	81	M	Unknown	Unknown	Nodule with an unclear boundary	10	Peripheral, LUL	Wedge resection	+	n.a.	n.a.
Ikeda^4)^	81	M	Unknown	Unknown	Nodule with an unclear boundary	10	Peripheral, RLL	Wedge resection	+	+	n.a.
	72	F	Unknown	Unknown	Nodule with an unclear boundary	5	Peripheral, LLL	Wedge resection	+	+	n.a.
Present case	76	M	+	−	Nodule with an unclear boundary	22	Peripheral, right S6 to S2	Wedge resection→ segmentectomy	+	+	+

α-SMA, alpha smooth muscle actin; F, female; h-caldesmon, heavy caldesmon; LLL, left lower lobe; LUL, left lower lobe; M, male; n.a., not available; RLL, right lower lobe

Radiologically, SMH can closely mimic primary lung cancer. In our patient’s case, the lesion demonstrated interval growth and a poorly defined border on CT, and both of these findings were suggestive of malignancy. Although PET revealed minimal FDG uptake (SUVmax 1.0), the lesion’s progressive enlargement necessitated surgical resection. This underscores the limited sensitivity of PET imaging in distinguishing rare benign entities such as SMH from early-stage malignancies.

Unlike inflammatory nodules, which are typically stable or regress over time,^9)^ SMH may demonstrate gradual growth. This characteristic can contribute to misdiagnoses and unnecessary invasive intervention. Clinicians should consider SMH in the differential diagnosis of slowly enlarging peripheral nodules, particularly in patients with chronic lung disease or a history of smoking.

The present case demonstrates the complexity of diagnosing pulmonary SMH both radiologically and pathologically. Intraoperatively, the tumor was initially suspected to be adenocarcinoma based on a frozen-section analysis, which revealed irregularly distributed atypical cells forming mildly irregular glandular structures. However, a retrospective review suggested that these glandular structures were likely attributable to the proliferation of type II pneumocytes in response to inflammation rather than true adenocarcinoma. The diagnostic challenge was further compounded by the extreme rarity of pulmonary SMH and the inability to perform immunohistochemical staining intraoperatively, which limited the accurate assessment of smooth muscle proliferation during surgery.

## CONCLUSIONS

SMH is an extremely rare benign disease that can mimic lung cancer and may be considered among the possible differential diagnoses of solitary pulmonary nodules. Given the diagnostic uncertainty, surgical resection is often necessary; however, limited approaches such as wedge resection or segmentectomy should be prioritized to minimize the possibility of overtreatment. Increased awareness and accumulation of SMH case reports are essential to improve the diagnostic accuracy and guide the optimal management of this rare condition.

## DECLARATIONS

### Availability of data and materials

All of the data in this article are available within the article.

## References

[1] Katzenstein A. Diagnostic atlas of non-neoplastic lung disease: a practical guide for surgical pathologists. New York: Demos Medical; 2016:48–75.

[2] Fraire AE, Dail DH. Mesenchymal tumors, Part I: Tumors of fibrous, fibrohistiocytic, and muscle origin. In: Tomashefski JF, Cagle PT, Farver CF, et al, eds. Dail and Hammar’s pulmonary pathology, 3rd ed. New York: Springer; 2008:427–442.

[3] Shikata S, Ueda Y, Kanno M, et al. A case of smooth muscle hyperplasia of the peripheral bronchiole revealed by video-assisted thoracic surgery after CT-guided marking of the small peripheral nodule (in Japanese). J Jpn Assoc Chest Surg. 2005; 19: 778–81.

[4] Ikeda T, Go T, Kadota K, et al. Two cases of nodular smooth muscle proliferation suspected of primary lung cancer from preoperative images: a case report. J Cardiothorac Surg 2020; 15: 179. 32698831 10.1186/s13019-020-01228-6 PMC7376911

[5] Zhu X, Chen WB, Xing FB, et al. Treatment, pathological characteristics, and prognosis of pulmonary inflammatory myofibroblastic tumor: a retrospective study of 8 cases. Front Oncol 2022; 12: 840886. 36059625 10.3389/fonc.2022.840886 PMC9428495

[6] Chen W, Lu C, Hirota C, et al. Smooth muscle hyperplasia/hypertrophy is the most prominent histological change in Crohn’s fibrostenosing bowel strictures: a semiquantitative analysis by using a novel histological grading scheme. J Crohns Colitis 2017; 11: 92–104. 27364949 10.1093/ecco-jcc/jjw126

[7] Barton JH, Davis CJ Jr, Sesterhenn IA, et al. Smooth muscle hyperplasia of the testicular adnexa clinically mimicking neoplasia: clinicopathologic study of sixteen cases. Am J Surg Pathol 1999; 23: 903–9. 10435559 10.1097/00000478-199908000-00007

[8] Kay JM, Kahana LM, Rihal C. Diffuse smooth muscle proliferation of the lungs with severe pulmonary hypertension. Hum Pathol 1996; 27: 969–74. 8816894 10.1016/s0046-8177(96)90226-9

[9] Zhang R, Tian P, Qiu Z, et al. The growth feature and its diagnostic value for benign and malignant pulmonary nodules met in routine clinical practice. J Thorac Dis 2020; 12: 2019–30. 32642104 10.21037/jtd-19-3591 PMC7330364

